# Creutzfeldt-Jakob Disease: Insights from a Case Report and Diagnostic Challenges

**DOI:** 10.7759/cureus.96375

**Published:** 2025-11-08

**Authors:** Harikrishna Choudary Ponnam, Kunal Sonavane, Pallavi Shirsat, Ragini Gopagoni, Laxmi Sakamuri, Rahul Kashyap

**Affiliations:** 1 Internal Medicine, Summa Health, Akron, USA; 2 Internal Medicine, Willis Knighton Medical Center, Bossier City, USA; 3 Nephrology, Minden Medical Center, Minden, USA; 4 Pulmonology, Mayo Clinic, Jacksonville, USA; 5 Research, WellSpan Health, York, USA

**Keywords:** creutzfeldt-jakob disease, csf protein 14-3-3, electroencephalography (eeg), prion diseases, rapidly progressive dementia

## Abstract

Creutzfeldt-Jakob disease (CJD) is a rare, rapidly progressive, and fatal neurodegenerative disorder caused by abnormal prion proteins. Due to its often nonspecific and variable early symptoms, CJD is frequently misdiagnosed as a psychiatric or other neurodegenerative condition, creating significant diagnostic challenges. Timely recognition is crucial but remains difficult due to its rarity and overlap with more common disorders.

We describe a 66-year-old male patient who was initially admitted to a psychiatric unit with prominent behavioral disturbances and mood changes. The clinical presentation was initially attributed to a primary psychiatric illness, particularly bipolar disorder. Brain imaging and electroencephalogram (EEG) were inconclusive. However, cerebrospinal fluid (CSF) analysis revealed findings consistent with prion disease, confirming the diagnosis of CJD. The patient’s neurological function deteriorated rapidly, and he died within two months of diagnosis.

This case highlights the diagnostic complexity of CJD, especially in its early stages when psychiatric symptoms may predominate. A high index of suspicion and early use of CSF biomarkers are essential for timely diagnosis. Clinicians should consider CJD in the differential diagnosis of rapidly progressive neuropsychiatric syndromes, as early recognition, although not curative, can guide appropriate care and family counseling.

## Introduction

Creutzfeldt-Jakob disease is a rare, rapidly progressive, fatal neurodegenerative disease that occurs due to the accumulation of misfolded prion proteins in the brain. It primarily affects the central nervous system with a wide range of neurological manifestations like neuropsychiatric symptoms, behavioral, pyramidal, and extrapyramidal symptoms. motor dysfunction. The incidence of the disease is one in a million [1]. Diagnosis is challenging due to the rarity of the disease and its presentation, which closely mimics other conditions such as autoimmune, vascular, and infectious etiologies, making it easy to overlook. Creutzfeldt-Jakob disease (CJD) can occur in sporadic, genetic, or acquired forms [2]. Sporadic CJD accounts for approximately 85-90% of cases, while genetic forms represent 10-15%, and iatrogenic and variant forms each account for less than 1% [3]. Here we present a case of CJD with progressive neurological decline and dementia.

## Case presentation

A 66-year-old male patient presented to the emergency department with persistent mixed delusions, specifically believing vehicles posed imminent danger. According to his sister, he was exhibiting a fixed and intense worry about an ongoing war in Gaza. Additionally, he experienced multiple falls, some of which resulted in head injuries; details of the extent of injury were not documented in medical records. He denied any suicidal ideations.

There was no reported history of consuming infected beef or a family history of CJD. On admission, his vital signs were stable: blood pressure was 114/57 mmHg, heart rate was 68 beats per minute, respiratory rate was 18 breaths per minute, temperature was 36.7°C, and body mass index (BMI) was 22.8 kg/m². On neurological examination, the patient was alert and oriented to person, place, and time but appeared very anxious and exhibited grandiose behavior. Comprehensive neurologic examination was within normal limits.

Initially, the patient was admitted to the psychiatry unit with a working diagnosis of bipolar disorder due to his mood symptoms and behavioral presentation. However, as the clinical picture evolved, concerns were raised about potential neurological causes, prompting further investigation for vascular and infectious etiologies. Comprehensive laboratory workup was conducted, including tests for Lyme disease, human immunodeficiency virus (HIV), anti-neutrophil cytoplasmic antibody (ANCA), syphilis (VDRL), thyroid-stimulating hormone (TSH), and autoimmune encephalitis. All results were negative. An electroencephalogram (EEG) did not reveal any abnormalities.

**Figure 1:**
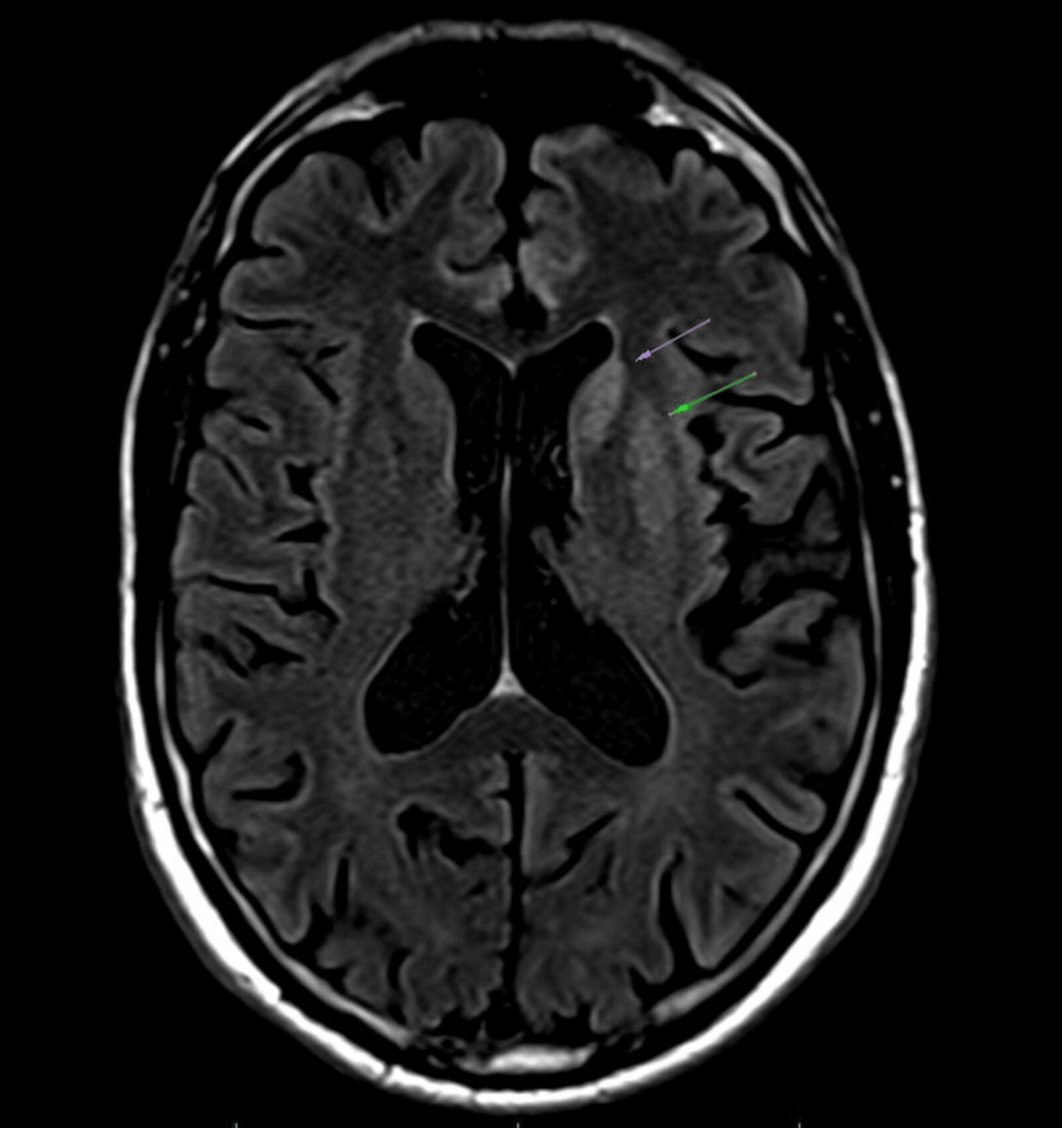
MRI showing T2 flair in the left hemisphere, precisely in the left basal ganglia, highlighted by the arrows

**Figure 2:**
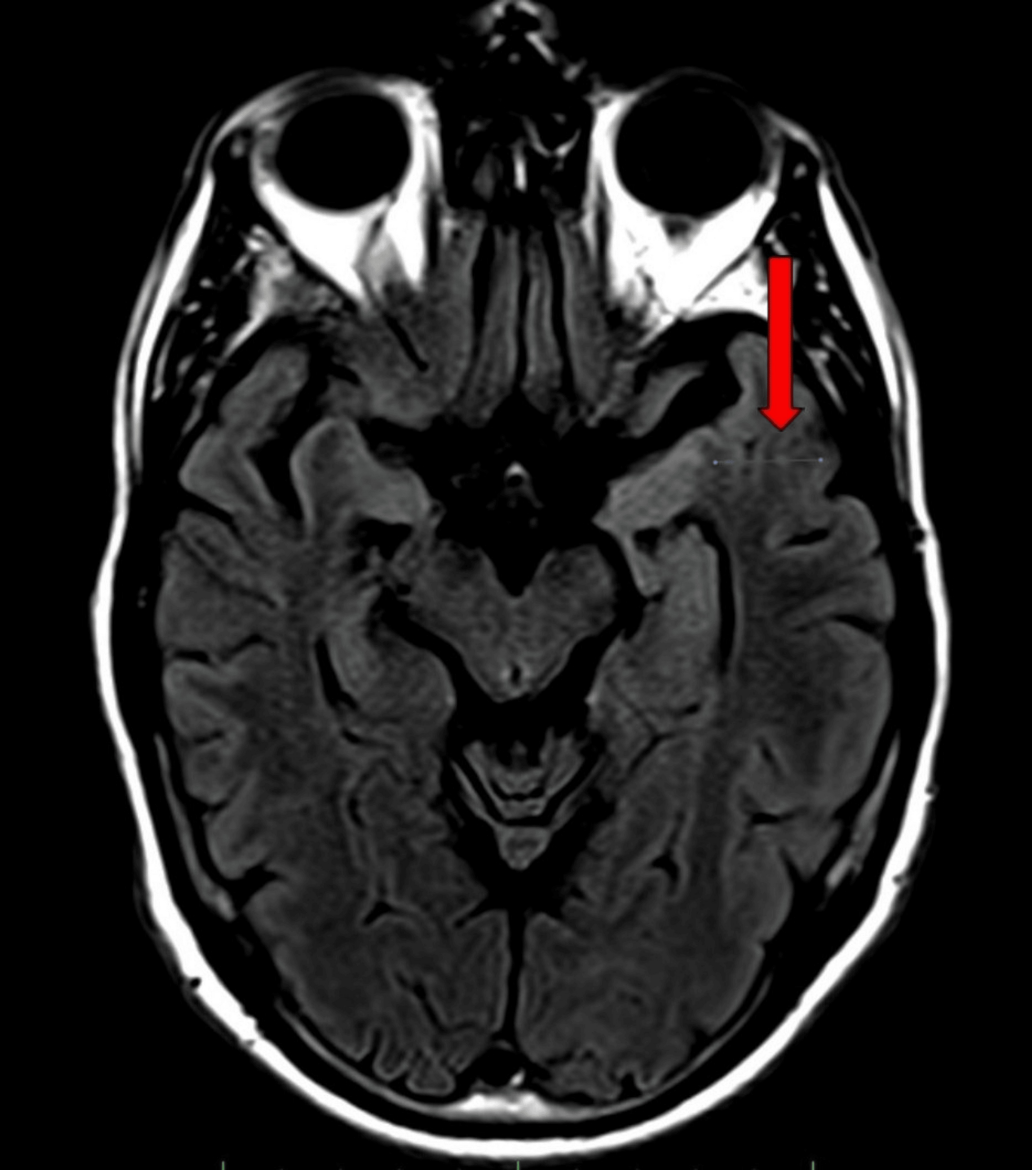
Diffusion weighted MRI imaging showing T2-flair in the left anteromedial temporal lobe, highlighted by the red arrow

Magnetic resonance imaging (MRI) revealed areas of restricted diffusion and fluid-attenuated inversion recovery (FLAIR) hyperintensity localized to the left basal ganglia and the left anteromedial temporal lobe, raising suspicion of a neurodegenerative or prion-related process (Figures 1, 2). The CSF analysis tested positive for the prion (14-3-3) protein, indicating rapid neurodegeneration. Over time, the patient’s condition progressively deteriorated. He became increasingly disengaged, exhibited rapidly worsening memory deficits, and developed significant impairments in both fine and gross motor functions. After consultation with the family, the patient was placed on hospice-level care. He was discharged to a nursing facility, where he passed away approximately one month later.

## Discussion

Creutzfeldt-Jakob disease (CJD) presents a significant diagnostic challenge due to its rarity and highly variable clinical manifestations, which can include rapidly progressive dementia, pyramidal and extrapyramidal symptoms, behavioral and personality changes, mood disturbances, visual hallucinations, ataxia, myoclonus, and speech abnormalities. Accurate diagnosis requires a high index of suspicion, as CJD is often easily overlooked or mistaken for other neurological disorders. Table 1 illustrates MRI findings in neurodegenerative disorders. Early differentiation is crucial for avoiding unnecessary treatments and reducing the burden on patients and their families. A definitive diagnosis is typically confirmed postmortem through the detection of pathological prion protein deposition in brain tissue, using methods such as Western blot analysis [4].

**Table 1:** Highlights of clinical characteristics and imaging findings associated with Creutzfeldt-Jakob disease (CJD) and other neurodegenerative disorders

Neurodegenerative disorders	Clinical features	MRI findings
CJD	Rapidly progressive dementia, personality changes, involuntary movement disorders, and muscle wasting	MRI flair in the left hemisphere, precisely the left basal ganglia and left anteromedial temporal lobe, CSF analysis with 14 3 3 Protein in CSF
Alzheimer’s disease	Cognitive decline, memory loss, behavioral changes, and executive dysfunction	MRI shows diffuse cortical atrophy in the temporal or hippocampal region, showing amyloid beta protein and Tau protein in CSF
Meningoencephalitis	Confusion, stiff neck, headache, seizure	Signal intensity abnormalities in the basal ganglia, thalami, brain stem, and leptomeningeal enhancement
Corticobasal degeneration	Movement disorders, dementia, and visuospatial deficits	Asymmetrical cortical atrophy in peri-Rolandic, posterior frontal, and parietal lobes

Several limitations impede the definitive diagnosis, including the lack of access to specific CSF biomarkers such as the 14-3-3 protein assay. Additionally, cultural and ethical considerations often pose significant barriers to performing post-mortem autopsies, which remain the gold standard for confirming the diagnosis. In our case, definitive diagnosis via brain biopsy was not performed, as per the family’s wishes.

A systematic review done to evaluate the efficacy of medications used in CJD and their role in neurocognitive decline [5] hypothesized the use of flupirtine. It upregulates the BCL-2 proto-oncogene and has demonstrated a cytoprotective effect on neuronal cells. However, this needs to be confirmed in large-scale studies. Disease attributes like the rarity of the condition and high mortality rate make designing clinical trials in humans quite challenging. 

Recently, the prion-degrading abilities of natto kinase and lumbrokinase have been identified in ME7 scrapie-infected mice as well as sporadic CJD patients [6]. Additionally, scientists have developed a three-dimensional (3D) tissue model of CJD to evaluate potential treatments; however, the lack of a fully representative human CJD model remains a significant obstacle. Approaches such as gene silencing using RNA interference, CRISPR-Cas9 [7] mediated gene editing, and antisense oligonucleotides are being explored to reduce the production of the PrPSc (Scrapie prion protein) or to correct genetic mutations that predispose individuals to inherited forms of prion disease. A targeted gene therapy has been proposed recently for the prevention of individuals with hereditary prion disease [8]. Anti-PrP (Prion protein) monoclonal antibodies delay the spread of prion disease when integrated with PrP.

Cellular prion protein(c) in cell culture and animal experiments [9]. There is currently no cure for CJD, and it is invariably fatal. A study conducted by the European CJD Surveillance, involving 2,451 patients with sporadic CJD, found that the median survival time was five months, with 85.8% of patients dying within one year of symptom onset [10]. Treatment is primarily supportive diagnosis remains challenging due to its broad clinical overlap with other rapidly progressive dementias and neurodegenerative disorders.

## Conclusions

Creutzfeldt-Jakob disease is a rare and fatal neurodegenerative disease. This case highlights the diagnostic challenges of CJD, underscoring the importance of careful clinical correlation with laboratory and imaging studies to support the diagnosis. Early and accurate diagnosis is essential for distinguishing treatable causes of rapidly progressive dementia from untreatable ones, thereby helping to avoid inappropriate or potentially harmful treatments providing appropriate patient counseling, and optimal supportive care planning.
